# Drone-Based Localization of Hazardous Chemicals by Passive Smart Dust

**DOI:** 10.3390/s24196195

**Published:** 2024-09-25

**Authors:** Tino Nerger, Patrick P. Neumann, Michael G. Weller

**Affiliations:** 1 Federal Institute for Materials Research and Testing (BAM), Richard-Willstätter-Straße 11, 12489 Berlin, Germany; tino.nerger@bam.de (T.N.); patrick.neumann@bam.de (P.P.N.); 2 Department of Chemistry, Humboldt Universität zu Berlin, Brook-Taylor-Strasse 2, 12489 Berlin, Germany

**Keywords:** passive smart dust, remote sensing, drone, UAV, hazard, optical detection, chemosensor, pH indicator, paper-based sensors, confetti-like sensor, harmful chemicals, accidents

## Abstract

The distribution of tiny sensors over a specific area was first proposed in the late 1990s as a concept known as smart dust. Several efforts focused primarily on computing and networking capabilities, but quickly ran into problems related to power supply, cost, data transmission, and environmental pollution. To overcome these limitations, we propose using paper-based (confetti-like) chemosensors that exploit the inherent selectivity of chemical reagents, such as colorimetric indicators. In this work, cheap and biodegradable passive sensors made from cellulose could successfully indicate the presence of hazardous chemicals, e.g., strong acids, by a significant color change. A conventional color digital camera attached to a drone could easily detect this from a safe distance. The collected data were processed to define the hazardous area. Our work presents a combination of the smart dust concept, chemosensing, paper-based sensor technology, and low-cost drones for flexible, sensitive, economical, and rapid detection of hazardous chemicals in high-risk scenarios.

## 1. Introduction

Smart dust represents an innovative concept in microtechnology. It involves the development of tiny sensors, primarily microelectromechanical systems (MEMSs), to monitor various physical, chemical, or other environmental parameters [1,2]. These small sensors, which have been planned to be as tiny as grains of sand, would be equipped with microprocessors, various sensors, batteries, and communication modules and enable the autonomous collection and wireless transmission of data to central networks [3,4]. Potential applications of smart dust are manifold, ranging from environmental monitoring to military surveillance, and promise transformative impact in areas such as agriculture, pollution control, ecosystem monitoring, and management of risk scenarios or emergency situations.

The development of the smart dust concept is attributed to Kris Pister and Joe Kahn, along with their teams at the University of California, Berkeley, in the late 1990s. The US agency DARPA supported some of these projects. For quite a while, smart dust was seen as an innovative concept with highly disruptive potential [5]. In 2013, smart dust was identified as an innovation trigger by Gartner Inc., but no longer appears to be included in recent versions of Gartner’s hype cycle for emerging technologies. Despite the promising prospects, smart dust technology still seems to face major challenges, such as miniaturization, communication, limited energy supply, safety and environmental issues, and costs [6]. In addition, deploying such electronic devices raises critical questions regarding safety, privacy, and environmental impact. The practical distribution of smart dust in the field has not yet been discussed in detail, and these sensors would need to be recollected after use, which is unlikely to have been a high priority given the other serious technological problems of which the power supply was the primary challenge. The future of this sensing technology is being explored with a focus on battery-less systems to achieve energy-autonomous sensors [7]. These sensors may operate independently of traditional battery power by leveraging other energy sources, such as light and thermal gradients. This would reduce maintenance needs and extend operational lifetimes, particularly important for applications in environmental monitoring and industrial automation. The further development of optically powered smart dust systems may also bypass the battery problem and emphasize efficient energy conversion and signal processing to enhance performance [8]. The broad potential of smart dust technology is evident across numerous applications. From precision medicine and environmental monitoring to industrial automation and renewable energy maintenance, these tiny sensors are poised to revolutionize data collection and use. As described, however, there are still considerable technical challenges that have not yet been solved [9]. Large challenges must be addressed in energy management, system integration, and miniaturization to realize the full capabilities of future smart dust systems.

This study focused on developing small biodegradable cellulose-based chemosensors for precise drone-based localization of hazards and the demarcation of danger zones. This confetti-like material, the key to this form of passive smart dust technology, was optimized for optimal detection by drone-based optical camera systems while enabling the coverage of larger areas with low production costs and environmental sustainability.

Thymol blue was chosen as a proven pH indicator due to its high molar absorptivity and distinct color changes at different pH levels. A polymer additive was used to keep the indicator sufficiently long on commercial filter paper. The manufacturing process was upscaled to obtain a larger amount of the confetti-like material. Dye bleeding was able to be significantly reduced compared to commercial products. Data processing involved converting drone-captured images from red, green, and blue (RGB) to hue, saturation, and value (HSV) color space for better color analysis. A custom Python script was used, including a threshold filter that identified areas with spilled acid. This was accompanied by several subsequent data processing procedures like Gaussian blur to reduce noise and a clustering algorithm grouping data points, enhancing the visualization of spatial relationships. Additional field tests confirmed the system’s potential for rapid hazard localization. The passive smart dust localized acid spills outdoors using a drone-mounted camera. Processed images identified contaminated regions based on color values, showing the system’s compatibility with low-cost drones.

The main contributions of this work are as follows.

- Production of small cellulose-based paper disks with a diameter of 6 mm acting as passive sensor components optimized for hazard detection and localization by standard drone cameras.
- Development of a reproducible manufacturing process to produce the first passive smart dust using thymol blue as a colorimetric indicator and a polyvinyl acetate additive to slow down the leaching of the dye. This method maintains high absorbency with minimal dye leaching, ensuring environmental compatibility and potential for large-area application.
- Automated image processing via an implemented data processing routine, including image segmentation, threshold filtering, and cluster analysis, used for optical hazard detection and precise localization.
- Validation under real-world conditions at a testing site. Demonstration that low-cost drones can effectively localize hazardous substances from a safe distance without modifications, making the system cost-effective and easy to use in contaminated areas.

## 2. Description of the Passive Smart Dust Concept

### 2.1. Conventional Application of Drones for Hazard Detection

Fast and reliable information is essential for managing critical situations and accidents with exceptional risk potential. Drones with special equipment have been tested and used regularly for these applications [10]. In recent years, drones have become increasingly popular and have also shown great capabilities in different data acquisition methods in object identification, gas detection, or remote sensing of hazard-related disasters [11,12,13]. Since they are remotely controlled, the operator can stay safe while investigating the region of interest. Very powerful drones are commercially available for typical disaster management, such as earthquakes, floods, or bushfires [14,15,16]. However, for chemical or biological risks, the capabilities of drones are still quite limited. Obvious challenges include the high diversity of such risks, which often need specific sensors that are only available for very few compounds. Taking samples with a drone and bringing them to a collection point or central laboratory seems slow, relatively expensive, and unsuitable for delivering a sufficiently dense data mesh. Drones equipped with electrochemical, metal oxide semiconductor, or non-dispersive infrared in situ gas sensors face challenges due to their propulsion systems causing downwash and strong turbulence [17,18]. This affects the spread of the chemical plume and makes it difficult to measure gas or vapor concentrations. Remote gas sensors offer new possibilities without direct contact with hazardous materials [19]. Besides remote sensing using multispectral analysis or fluorescence, monitoring larger areas is still limited with conventional sensor technology. Passive smart dust addresses these challenges by using chemically sensitive dyes that provide localized information that can be read by a standard drone camera.

### 2.2. Paper-Based Analysis

Paper-based analytical techniques are quite old and have always been popular in some areas, such as pH testing or water analysis [20]. Some scientists considered this approach to be outdated due to rapidly developing and more powerful analytical instruments. However, these sophisticated and often expensive devices are neither mobile nor economically accessible to many potential users. Paper-based analysis has experienced an astonishing renaissance in recent years. It has developed into an independent branch of analytical chemistry, particularly in the context of extremely mobile point-of-care systems or diagnostics in developing countries. Today, the technology of paper-based sensors has already reached a high level of sophistication and performance [21,22]. Results are mostly presented as a simple optical response, e.g., in the well-known corona rapid tests. In response to the as yet unsolved challenges of conventional smart dust, one approach to passive smart dust is the application of small, confetti-like pieces of paper. They are coated with chemically reactive pH indicators, acting as chemosensors. Many chemosensors have been developed to interact selectively and quantitatively with target compounds, including metal ions and glucose as well as to detect explosives, thereby expanding their applications. These reagents, often relying on optical effects like fluorescence or color changes, can be read out manually or nowadays also by smartphone apps [23]. These target–dye combinations have the potential for developing additional variants of passive smart dust.

### 2.3. Detection of Chemical Hazards by Passive Smart Dust and a Drone

**Figure 1:**
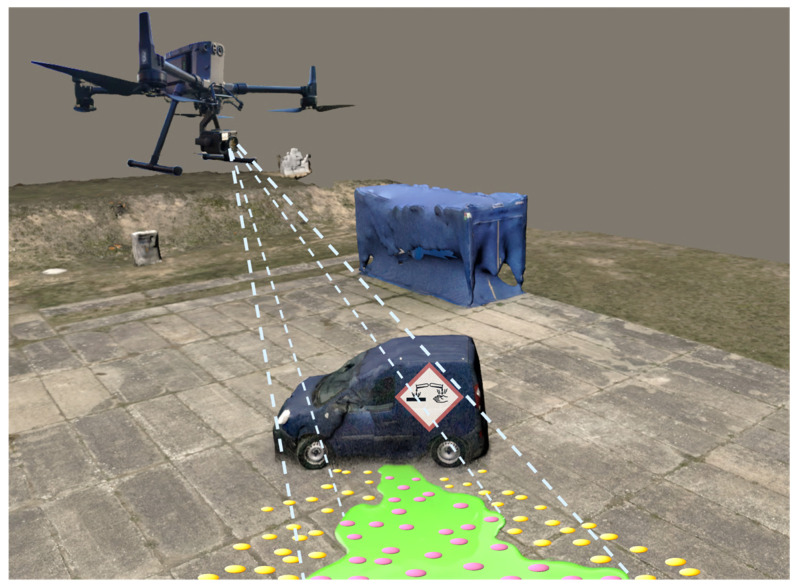
Schematic representation of the passive smart dust principle in the scenario of spilled liquids following an accident. The background image is a photogrammetric image of our test site. The hazardous liquid is shown in green for better visibility while being colorless in reality. The drone camera can identify and locate the hazardous area by a color change in our distributed passive smart dust.

The chemical information is read out by the color camera of a drone, also known as an unmanned aerial vehicle (UAV), which flies over the pieces of paper coated with colorimetric reagents at a certain distance. Passive smart dust indicates chemical hazards, such as with a color change, which can be recorded by any optical camera system attached to the UAV. Expensive multispectral cameras used in traditional remote sensing are not required. The benefits of such a system are versatility and low cost. In addition, the small pieces of paper used in this work are completely biodegradable, like other materials mainly based on cellulose. The scenario of an acid spill caused by a truck accident was chosen to demonstrate the passive smart dust concept (Figure 1). The well-defined color change of a pH indicator could be used as a proven and reliable method for detecting acids or bases. Small confetti-like pieces of paper were manufactured and tested in the laboratory. They were then deployed in an outdoor test area and analyzed using images from a drone camera hovering over the region of interest. The field test data were evaluated using a customized Python (version 3.9.5) program to detect and locate the contaminated area automatically. A color-based threshold filter was combined with additional criteria to create useful images of the hazardous region, e.g., for emergency services. 

## 3. Materials and Methods

### 3.1. Colorimetric pH Indicator

Thymol blue, 4,4′-(1,1-dioxido-3H-2,1-benzoxathiol-3-ylidene)bis [5-methyl-2-(1-methylethyl)-phenol, is a versatile pH indicator used in various applications, such as biology, analytical chemistry, and environmental science. It has high molar absorptivity, so it can be easily detected optically, even in low concentrations. This organic dye belongs to the subclass of the sulfonephthaleins and exists in three differently protonated forms [24,25]. These have three corresponding colors—purple, yellow, and blue—depending on the respective pH value. The suitability of a pH probe or indicator can be evaluated based on its acid dissociation constant, Ka (also referred to as acidity constant), or its negative decadic logarithm, pKa. In practical terms, the pKa value denotes the pH range over which an indicator changes its optical properties, making it useful for sensing applications [26,27]. The change in spectral properties of pH indicator dyes is caused by protonation or deprotonation. Thymol blue (CAS 76-61-9) offers the possibility of releasing two protons. Its lower pKa was determined to be 1.7, indicating that thymol blue converts to its fully protonated form (H_2_Ind) at a pH below 1.7, displaying a purple–red color (absorbance maximum at 546 nm). At a pH value between 1.7 and 8.9, thymol blue exists mainly in its singly protonated form (HInd^−^), which appears bright yellow (absorption maximum at 433 nm). At a pH higher than 8.9, it is present mainly in its blue and completely deprotonated form (Ind^2−^, absorption maximum at 600 nm) [28,29]. The absorption spectra were determined using an UV-vis spectrometer (NP80, Implen, Munich Germany). The ability to undergo a reversible color change over a relatively narrow pH range makes this dye suitable for titrations and other analytical techniques requiring visual pH measurements [30].

### 3.2. Manufacturing of pH-Sensitive Cellulose Sensors

**Figure 2:**
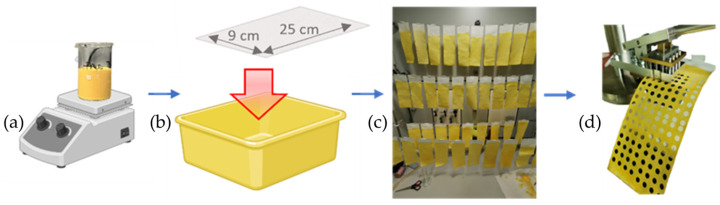
The production process of the colorimetric pH paper. (**a**) Starting with mixing the precursors and (**b**) immersing filter paper strips in a plastic tray containing the reagent mixture. The coated material was then dried by hanging it on a “washing line” (**c**). Finally, the coated cellulose chemosensors were punched out with a custom-made 6 mm Ø hole punch (**d**). Modified from BioRender.com.

Filter paper (MN 617, Macherey-Nagel, Düren, Germany) consisting of untreated cellulose was chosen as the raw material. 300 mg of thymol blue sodium salt (CAS 62625-21-2, ACS grade, Carl Roth, Karlsruhe, Germany) was dissolved in 400 mL of ultrapure water under stirring at 60 °C for 1 h. After the mixture cooled down to room temperature, 200 mL of Ponal^®^ super3 was added under constant stirring. Subsequently, the mixture was adjusted to a neutral pH by NaOH solution (50%, Honeywell, Raunheim, Germany) while monitoring this process with a pH electrode (HI5221-02, Hanna Instruments, Vöhringen, Germany). The resulting suspension was then transferred to a plastic tray, into which the filter paper strips (9 × 25 cm^2^) were immersed for 20 s each. Excess material was removed by pulling the wet paper strips through a small adjustable slit. Subsequently, the paper strips were then dried by hanging them on a “washing line” in a fume hood overnight at room temperature. Finally, the colorimetric sensor disks of 6 mm were cut out using a custom-made punch as shown in Figure 2.

### 3.3. Data Processing

In this study, visual data captured by a drone-mounted camera were stored as JPEG files and analyzed. The JPEG compression algorithm employs a discrete cosine transform (DCT) to convert spatial domain data into the frequency domain, followed by quantization and entropy coding for effective compression. This process balances image quality with file size, facilitating efficient storage and transmission. Decompression involves reversing these steps through entropy decoding, dequantization, and inverse DCT, restoring the image in the RGB color space with 8 bits per channel, suitable for being used in the displays of digital devices [31,32]. JPEG is a lossy format, which is not preferred for quantitative work. However, we were able to show that the data quality was sufficient for the purpose described. This has the advantage of even inexpensive digital cameras being able to be used to examine passive smart dust, and the files are also much smaller than those of lossless raw formats.

#### 3.3.1. Color Space Conversion

Due to the limitations of the RGB model in accurately rendering colors under varying lighting, we converted the recorded images into the HSV color space for enhanced color analysis. The HSV model categorizes colors in a cylindrical coordinate system based on their hue, saturation, and brightness levels [33]. The conversion process involves normalizing the RGB values, finding the maximum and minimum values, calculating the hue, saturation, and value based on the RGB components, and expressing the derived HSV parameters. The hue component, measured as an angle between 0° and 359°, corresponds to the color’s dominant absorption wavelength and provides a robust indicator of color regardless of lighting conditions. This is very beneficial for precisely detecting specific chemical hazards indicated by a specific colorimetric response. The saturation and value are set between 0 and 255 and set to a particular range to improve filter results. This approach significantly mitigates the variability introduced by the RGB model, thereby enhancing the reliability of the detection system in variable environmental contexts [34,35].

#### 3.3.2. Filtering Process

An HSV threshold filter was used to identify particles signaling acidic (or neutral) areas. The filter hue settings (Table 1) were adjusted based on the results of previous tests. To accommodate diverse outdoor conditions, mainly caused by different lighting, the boundaries of the HSV threshold filter were set wider than in a usual laboratory setup. After the threshold filtering, a Gaussian blur method was used to reduce image noise and smooth out variations. The function applied adaptive thresholding, making it useful for images with varying lighting conditions by individually calculating the threshold for small regions. This results in a binary image where different image parts have different thresholds, enhancing visibility and detail in unevenly lit photos. Thus, fragments not representing our sensor disks were excluded [36,37,38]. It also mitigates chromatic aberration effects due to the rolling shutter configuration of the camera platform used. Afterward, the density-based spatial clustering of applications with noise (DBSCAN) algorithm was used [39,40]. This algorithm helps identify clusters within the data by specifying the maximum distance between two points so that they can be considered part of the same cluster. It groups closely packed points while marking points in very-low-density regions as outliers. This algorithm is particularly useful for data with noise and varying density, identifying dense areas, finding arbitrarily shaped clusters, and removing outliers. Here, the radii of the circles drawn around the remaining data points, centered on the centroids of the points in the largest clusters, were also determined. This approach ensures consistency between the clustering process and the following visualization, providing a clear and accurate representation of the spatial relationships among the data points given by the threshold filter. The passive smart dust system’s resolution is based on the distribution of sensors on the ground. As such, a safety radius that is drawn around each sensor signaling acid is not just an heuristically chosen value, but a dynamic one depending on the distribution and distance between the sensors. This calculation was made to have a reasonable safety margin for first responders and obtain a visual output for decontamination actions.

### 3.4. Experimental Outdoor Setup

**Figure 3:**
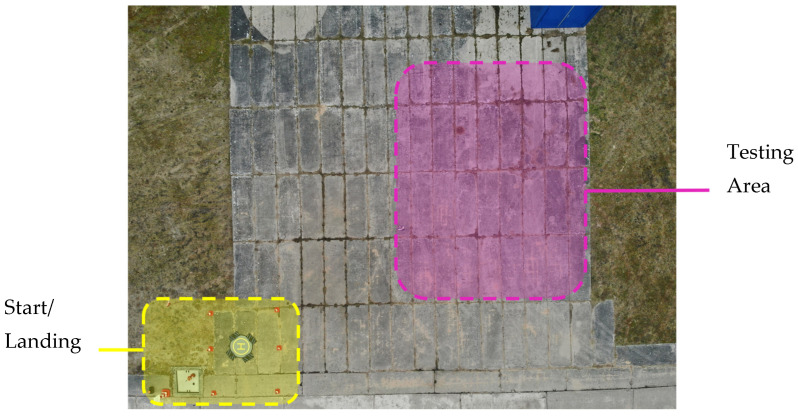
An overview of the testing site for the outdoor experiments. After takeoff, the UAV collected images while hovering above the region of interest (ROI) where the passive smart dust was manually distributed over the different test liquids.

**Figure 4:**
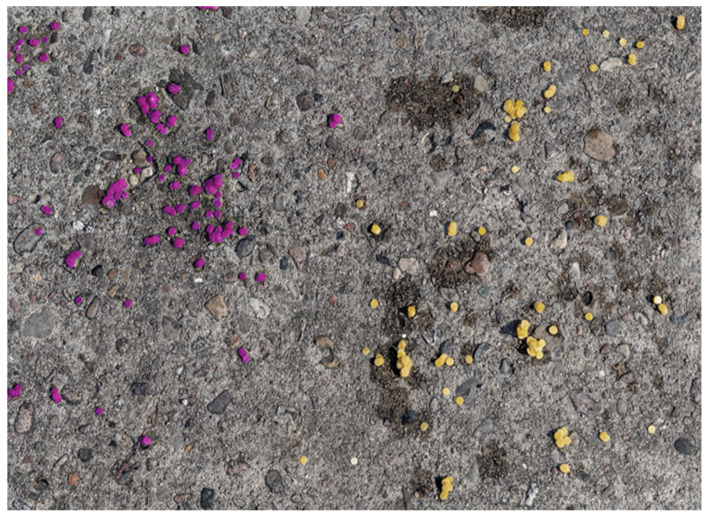
Colorimetric cellulose sensors on the testing ground. Red–violet points on the left after contact with concentrated hydrochloric acid (37% HCl) and yellow sensors on the right after contact with water. The liquids had already partially evaporated when this picture was taken. Photo courtesy of Julia Päpke (BAM).

Weathered concrete slabs (Figure 3) were used as a realistic surface for the field tests. In Figure 4, a close-up can be seen and shows a material mix, which is even more demanding for threshold filtering compared to a more homogeneous background. The density and even distribution of the paper sensors is decisive for the spatial resolution of the results. In this preliminary study, the confetti sensors were spread manually by releasing them from a height of about one meter. First the whole area was uniformly moistened to obtain an even wet surface on which acid spills could not be localized based on the edges of the wetness. Subsequently, the concentrated acid was spread in the center using a sprayer. The whole area simulating the spilled acid was around 15 m^2^. An overflight altitude of 20 m with a 10× zoom level of the drone camera was finally chosen, resulting in a reasonable image resolution while not having the passive smart dust affected by the rotor downwash. With these settings, a paper disk of 6 mm diameter has a resulting size of about 255 pixels, which allows excellent statistical evaluation, but also shows the potential for a further reduction in the size of the paper disks. All images were stored in the JPEG format and contained geolocation data in exchangeable image file format (Exif). Due to the use of a real-time kinematic (RTK) transmitter module for positioning, these data provide the exact location in a centimeter range.

### 3.5. Drone Platform

**Figure 5:**
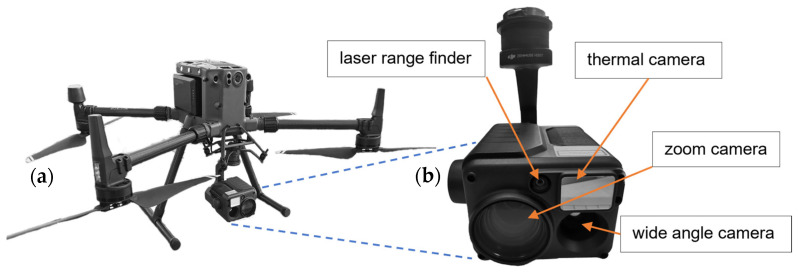
DJI Matrice 300 RTK drone system (**a**), including payload, and the Zenmuse H20T multi-sensor camera housing used for the experiments (**b**).

In our study, we used the drone DJI Matrice 300 RTK (Figure 5a). It is a commercially available quadcopter made by Da-Jiang Innovations Science and Technology Co., Ltd., Hangzhou, China. It is suitable for operation in harsh weather conditions. An RTK ground station was used to determine precise localization data. The drone offers a maximum flight time of 55 min with a range of up to 15 km. A payload of up to 2.7 kg can be attached to the platform. A DJI Zenmuse H20T (Figure 5b) was used for optical measurements. The H20T is gimbal-stabilized, which is important for obtaining high-quality images, and it contains a zoom-capable 20-megapixel (MP) 1/1.7″ complementary metal–oxide–semiconductor (CMOS) sensor and a 12 MP wide-angle 1/2.3″ CMOS sensor. Moreover, an attached VOx microbolometer can measure wavelengths in the near-infrared range, and a 905 nm class 1M laser provides relevant distance information up to 1.2 km [41]. For the optical detection of the passive smart dust particles, the 1/1.7″ sensor was used. This has an active area of 7.41 mm × 5.56 mm with a pixel size of 2.04 µm^2^, resulting in 20.15 MP in a 4:3 format.

## 4. Results

### 4.1. Colorimetric Confetti-like Sensors

**Figure 6:**
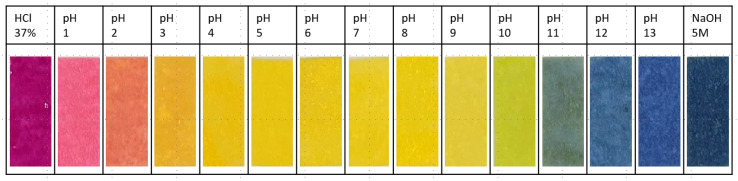
Polymer dye mixture color reaction on buffer solutions with different pH values, starting from extremely low pH on the left up to high values shown on the right.

**Figure 7:**
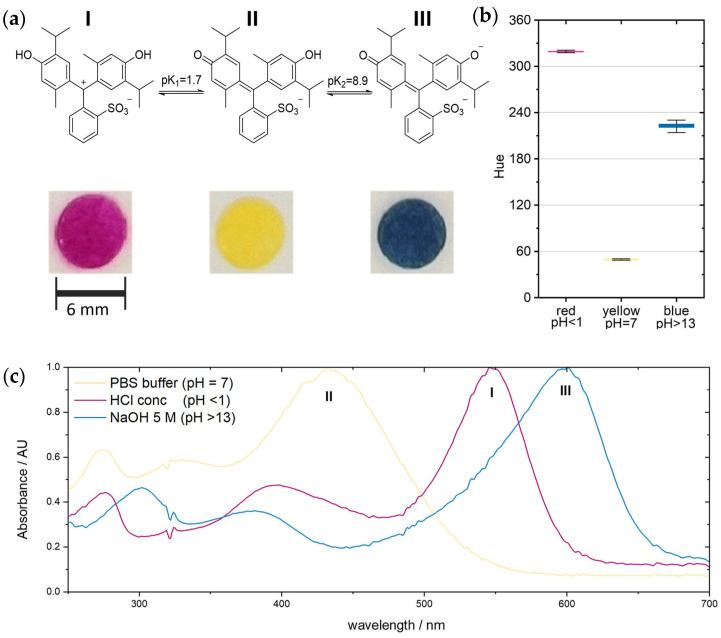
The three different forms of thymol blue and corresponding colorimetric sensor of 6 mm diameter (**a**). Recorded deviations of the hue (based on 20 samples with 1 million pixels) for each color (**b**). Normalized absorption of thymol blue depending on the surrounding pH (**c**).

**Figure 8:**
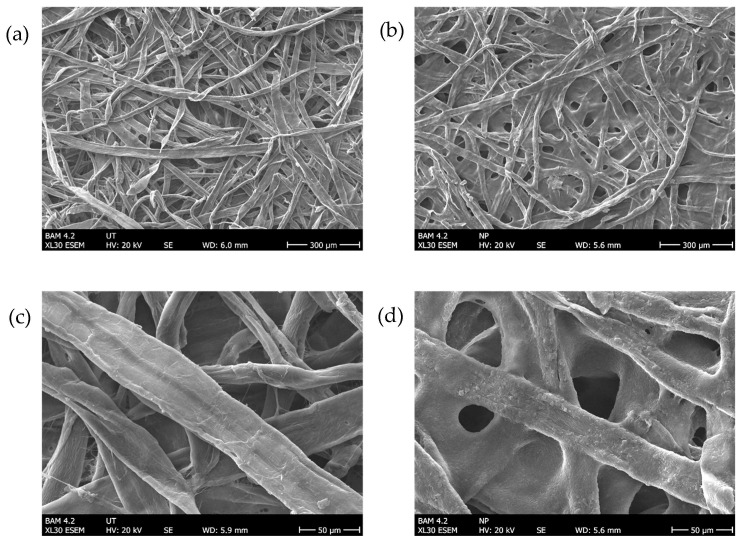
ESEM micrographs of the raw cellulose material (**a**,**c**) and the resulting colorimetric sensor treated with the described polymer dye mixture (**b**,**d**). Courtesy of Ines Feldmann (BAM).

The manufactured paper sensors can detect hazardous acids and bases reliably. For the application of a leakage, only the color reaction to extreme pH values is needed, and a color chart for each pH can be seen in Figure 6. For dependable detection using a color filter, a minimal hue deviation (approximately synonymous with the perceived color) was achieved after optimizing the manufacturing process, as shown in Figure 7b. Close-up shots of 20 samples of each color were recorded after contact with HCl (37%), water, and 5 M NaOH. Of these 20 samples, 10 were from one production run and 10 were from a second batch (Table 2). As the manufacturing process is still on a laboratory scale, different batches may reveal potential reproducibility problems. The average hue of 1 million pixels of each sample was then calculated and shown in Figure 8c. Small error bars for the obtained base material and after contact with concentrated hydrochloric acid show a reliable hue. The larger deviation in the hue after being in contact with sodium hydroxide is caused by divergences in the second production run, which is underlined by the values found in Table 2.

**Figure 9:**
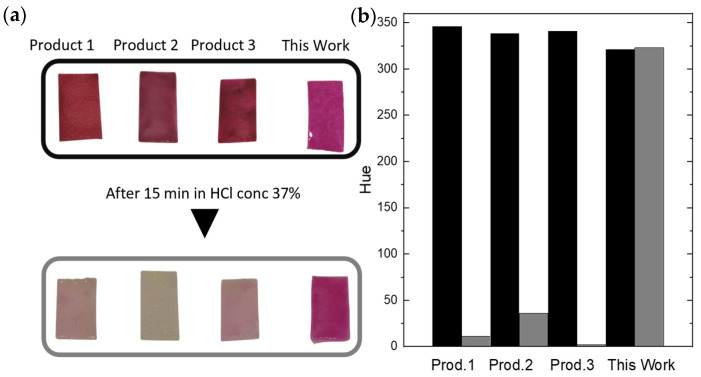
The dye bleeding of our product compared to three commercial products after 15 min exposure to concentrated hydrochloric acid (**a**). The before-and-after hues are shown in a bar chart (**b**). For the passive smart dust application, a stable hue is preferable.

Adding the Ponal polymer led to a sufficient reduction of dye bleeding compared to other commercially available pH strips. Figure 9 shows the stability of the hue of our product compared to three commercial products after being immersed in HCl (37%) for 15 min. In a scenario where a drone first deploys the sensors over a certain area and afterward hovers over this region and scans for the colorimetric reaction, it is beneficial that the color stays stable enough, at least over this period. Also, the water resistance and mechanical properties of filter paper can be enhanced with the addition of a polymer [42]. We observed the bleeding over a period of several hours, and after one hour, 35% of the dye used was still present on the sensor material. The reduced porosity and increased smoothness of the paper surface gave the final sensors a glossier appearance compared to a raw cellulose dye combination. Figure 8 shows the structural changes to the cellulose carrier material caused by the polymer additive using an environmental scanning electron microscope (ESEM). Adequate porosity of the material was maintained to allow the sample liquid to be sufficiently absorbed by the sensor material. The cellulose fibers were covered with the used polymer dye mixture after drying. The polyvinyl acetate dye mixtures provided an accurate and cost-effective method for measuring strong pH changes, as the encapsulated pH indicator dyes maintained their color-changing properties when embedded in a polymer [43,44,45].

### 4.2. Optical Detection of Hazardous Liquids

**Figure 10:**
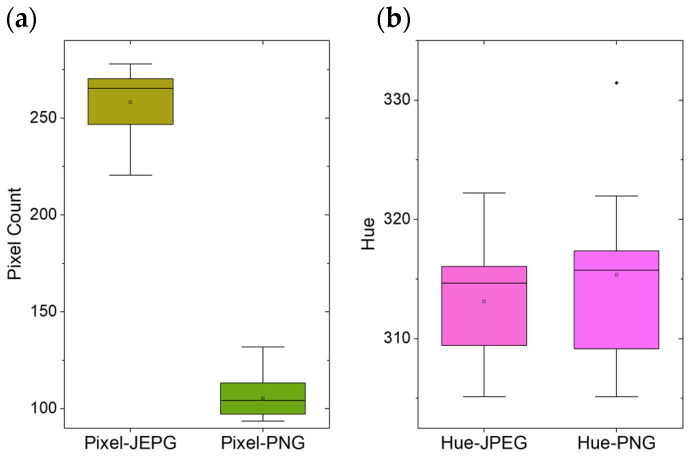
A comparison of compressed (JPEG) and lossless (PNG) image data of the 6 mm-diameter disks used as passive smart dust. (**a**) The pixel number associated with the cellulose disks taken from the drone at 20 m height. (**b**) A comparison of the average hue of those areas revealed only a minor difference in the format of image storage, making standard drone cameras a suitable option.

**Figure 11:**
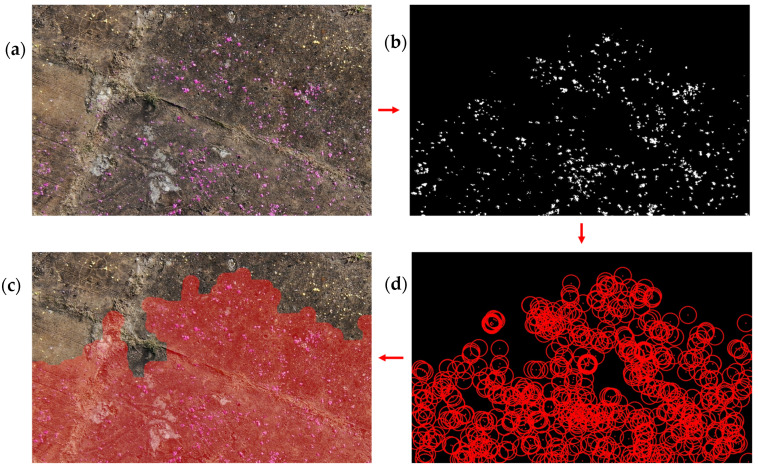
(**a**) Close-up of the drone footage from the testing area. (**b**) The applied threshold filter displays all sensor disks, indicating the hazardous zone, and in this case, the presence of strong acid. (**c**) An optical representation combining gathered information with image material to localize the hazardous region as a red area. (**d**) The reduced dataset after DBSCAN (as described in 3.3.2), with distance base safety radii drawn around the positive sensor disks.

Images of the ROI were processed by a custom script written in Python (https://github.com/BAMresearch/PassiveSmartDust.git). After converting the data to the HSV color space, a threshold filter was used. This combines color information with additional criteria, as described in Section 3.3.2. After this procedure, all areas define the contaminated region based on their previously determined color value, which indicates the corresponding hazard. The color filter settings for the lower and upper range of the HSV parameters were helpful in compensating for some variations in the color value. As mentioned, the decompression in JPEG image data results in the formation of some image artifacts. However, as our concept should apply to as many commercially available drones as possible, we have also investigated how much JPEG images and lossless formats such as PNG differ [46]. The results of these tests showed that the number of positive pixels differed considerably (Figure 10a). The drone took images at a height of 20 m above the ROI. Our 6 mm-diameter paper sensors were then extracted from JPEG and PNG images. The pixel number associated with the sensors was significantly higher in the JPEG images. This is mainly due to blurred edges [47,48]. However, when examining the average hue values, hardly any differences could be observed between the two tested image formats. This result clearly shows that the color-based detection and localization of hazardous substances is also possible with simple drone cameras in the low-cost range, which often use lossy compression algorithms to store the image data. The colorimetric reaction of the thymol blue dye has also been well investigated. The shift in absorbed wavelength between the three different forms (H_2_Ind, HInd^−^, Ind^2−^) and the corresponding hue number is large enough to visually detect higher pH changes on a color basis. This could be successfully examined with a drone-based optical camera system from a distance, as shown in Figure 11. Here, the DJI Matrice 300 RTK was chosen for its robust capabilities, and the attached Zenmuse H20T camera, with its stabilized lens system, was able to take sharp photos in various weather conditions.

The photos’ Exif data provided the potential hazard’s exact location. As an RTK GPS was used in the tests, highly precise location data in the centimeter range could be obtained [49]. For ease of use on-site, the positively displayed test areas were also visualized in Google Earth using the KML (keyhole markup language) file format.

## 5. Discussion

### 5.1. Application of Passive Smart Dust for Hazard Localization

Inexpensive “confetti sensors” were produced using cellulose filter paper with the immobilized indicator dye thymol blue. A manufacturing process was developed based on reproducibility tests and the examination of different polymer dye and paper combinations. Testing of the color values before and after reacting with hydrochloric acid as a model compound of a chemical hazard was first performed in the lab and then transferred to the field. Here, it was analyzed whether simple drones were suitable for this purpose. The lossy JPEG image format was tested against a lossless format and proved sufficient to obtain reliable data, described as hue color values. The color space transformation was chosen since when filtering for a specific color in RGB, the definition of thresholds for each of the three channels is required, and variations in lighting can affect the color’s perceived values. By focusing on the hue of a specific dye, the examination of specific colors is effective regardless of their intensity and saturation. This anticipates the changing environmental conditions in field trials. The drone’s flight speed was limited to 1 m/s to reduce distortions caused by the rolling shutter of the camera sensor. A testing site with an area of 15 m^2^ was used for a small but realistic accident scenario. As described, different heights of 10 m up to 50 m were tested in combination with several zoom levels, from 5 to 20, of the drone camera. Regarding image data processing, a custom script in Python was chosen because of the huge variety of libraries and capabilities in computer vision [50,51]. The chosen safety radius around each positive signal was used instead of a simple fixed value, since the resolution of the system depends on the distribution of the paper sensors in the area of interest.

Compared to various commercially available pH papers, thymol blue immobilized on cellulose paper by a polymer additive was significantly more stable regarding color bleeding. The laboratory experiments showed reproducible color values before and after exposure to strong acids. The JPEG format in which the captured image data were stored uses compression algorithms that result in some loss of information. Surprisingly, the effects of the JPEG algorithm with quantization and subsequent decompression had only a very small influence on the average hue values used for the segmentation filtering. The drone camera with a stabilizing gimbal and the standard lens system provided valuable image data.

Laboratory tests and field tests at the BAM test site (Horstwalde) confirmed the potential for rapid and effective hazard localization. Regarding the environmental impact of the paper pieces, it can be inferred that all materials and chemicals used should be sufficiently biodegradable. Therefore, in most cases, the paper sensors can remain in the environment and be left to natural degradation; however, this still needs to be confirmed experimentally. In most cases, recovery and disposal of the used sensors should be unnecessary. In the event of an accident involving a contaminated area, this zone must be neutralized and cleaned anyway.

### 5.2. Limitations and Improvements

In addition to the indicator dye thymol blue, other pH indicators and polymers were also examined. Polyethyleneimine, polyethylene glycol, or chitosan combined with some dyes were tested. They proved inferior to the combination of thymol blue and Ponal, which achieved the best results in bleeding inhibition and color reflection. While targeting a high-risk scenario with extreme pH values, the system may be improved to recognize smaller changes in pH when a mixture of indicator dyes is used. The covalent binding of the indicator dye to cellulose would eliminate the need for polymer additives [52]. Cellulose was chosen for its good availability and biocompatibility. Long-term monitoring of areas of interest may also be an interesting option. In this case, more stable chemosensor materials might be required.

The automated stitching of several images into one image would be necessary to examine large areas. This option might be included in future software versions of some drones or using telemetry data and third-party software, as used in photometry. Our system works in daylight but was not tested in the dark. However, the chosen drone offers the possibility of a dual-gimbal mount, which can be used simultaneously with a strong spotlight and a camera. Furthermore, fluorescent dyes, which have been tested in preliminary experiments and are known for their applications in many other applications, could be used as chemosensors. Although our approach has proven reliable and produces qualitative results, we aim to develop more quantitative and selective methods.

The deployment of the acid- or base-indicating passive smart dust over the area of interest was still undertaken manually to explore the system in a research environment. However, other options must be investigated to deliver a robust, fully integrated system to fulfill the real remote detection task. Passive smart dust is not limited to the used quadcopter: other systems, such as blimps, are conceivable. Here, however, we have focused on an application for first responders. Due to the rapid development in this field, it is conceivable that every fire engine will be equipped with drones in the coming years.

Finally, data processing could be significantly improved to better distinguish between paper sensors and interfering backgrounds, including various surfaces such as asphalt, concrete, gravel, vegetated land, and more. In addition, different environmental conditions, such as lighting, fluctuations in wind speed, and precipitation, such as rain and snow, must be investigated.

### 5.3. Future Research

In the next steps, other hazardous compounds will be addressed to obtain a larger portfolio of substances that can be detected. First experiments with different color-changing materials providing fluorescent emission have been successfully performed. Adaptation to gases or vapors could open an even wider range of applications. The drone’s flexibility allows the attachment of conventional sensors for gas detection. In combination with the information provided by the passive smart dust, gas leakage on the ground could be detected and localized. Additionally, combining data obtained by passive smart dust with the thermal imaging camera in the drone could lead to a useful fusion of multidimensional data.

In addition, systems for releasing passive smart dust directly from the drone and other options, such as delivery with robotic vehicles or launching of confetti cannons, should be investigated. As mentioned earlier, drones and machine learning algorithms will be developed for faster and more accurate threat detection. Building on the initial results for fast image recognition, this direction will be the future for segmentation and detection algorithms.

## 6. Conclusions

The passive smart dust concept significantly advances the economical and effective remote detection and localization of hazardous substances. This innovative interdisciplinary approach used paper-based confetti-like sensors impregnated with indicator dyes that can be remotely deployed and analyzed using commercially available drones equipped with optical cameras. After process optimization, the manufactured particles maintained a consistent hue with minimal deviation, as evidenced by close-up images of samples exposed to concentrated HCl. The average hue, calculated for two different production runs, confirmed the product’s reproducible properties. Compared to commercially available pH papers, the use of polymer additives significantly reduced dye bleeding.

Drone integration further enhanced the system’s utility and opens the door for safe and remote deployment of sensors. Image processing involved converting data to the HSV color space and applying threshold filters to identify contaminated regions. Comparisons between JPEG and PNG formats revealed the minimal impact of JPEG compression on hue values, underscoring the reliability of color-based detection even with low-cost drone cameras.

Nevertheless, some limitations and areas for improvement were identified. While the manual distribution of sensor confetti is suitable for research purposes, automated deployment methods need to be developed in the future, e.g., drone delivery systems, other robotic vehicles, or a device such as a confetti cannon. The system’s effectiveness in low-light conditions or even darkness is possibly limited and has not been tested. Future enhancements could incorporate fluorescent dyes or dual-gimbal mounts with strong spotlights for nighttime operation. We are already working on improved data processing algorithms essential for distinguishing sensor disks from complex background surfaces, including deep learning models. Additionally, expanding the range of detectable hazardous substances, including gases and vapors, would enhance the system’s utility.

In a nutshell, the passive smart dust concept represents a new approach to the remote detection of chemical hazards. The system may offer a practical solution for emergency response teams and other applications requiring rapid situational assessment by utilizing low-cost, readily available materials and standard drone technology.

## Figures and Tables

**Table 1:** Boundaries of the threshold filter settings used. A range of around ±30 was added for the average hue determined experimentally in the lab to balance natural variations. Adjustments in the saturation and value were added to reduce other noise, allowing a better segmentation of the passive smart dust of the background.

	Lower Limit	Upper Limit	Range
Hue	293	356	0–359
Saturation	95	213	0–255
Value	73	255	0–255

**Table 2:** Hues of ten samples for two batches each. Both batches provided consistent hue numbers after contact with HCl 37% and water. Here, the second batch showed larger deviations in hue after being in contact with NaOH 5 M (far right of table).

	Hue pH < 1 1st Batch	Hue pH < 1 2nd Batch	Hue pH = 7 1st Batch	Hue pH = 7 2nd Batch	Hue pH > 13 1st Batch	Hue pH > 13 2nd Batch
Mean	319.01	319.96	49.15	50.12	224.11	220.04
SD	0.582	1.542	0.378	0.225	2.168	8.594

## Data Availability

The Python code scripts used to analyze the image datasets are available on GitHub: https://github.com/BAMresearch/PassiveSmartDust.git.

## References

[1] Pister K.S.J. (2001). Smart Dust: The Autonomous Sensing and Communication in a Cubic Millimeter. Computer.

[2] Shaik M., Shaik N., Ullah W. (2016). The Wireless Sensor Networks: Smart Dust. Int. Res. J. Eng. Technol..

[3] Sailor M.J., Link J.R. (2005). “Smart Dust”: Nanostructured Devices in a Grain of Sand. Chem. Commun..

[4] Culler D., Estrin D., Srivastava M. (2004). Overview of Sensor Networks. Computer.

[5] Doherty L., Warneke B.A., Boser B.E., Pister K.S.J. (2001). Energy and Performance Considerations for Smart Dust. Int. J. Parallel Distrib. Syst. Netw..

[6] Farooqui M.F., Karimi M.A., Salama K.N., Shamim A. (2017). 3D-Printed Disposable Wireless Sensors with Integrated Microelectronics for Large Area Environmental Monitoring. Adv. Mater. Technol..

[7] Hester J., Sorber J. The Future of Sensing is Batteryless, Intermittent, and Awesome. Proceedings of the 15th ACM Conference on Embedded Network Sensor Systems.

[8] Liu J., Zhou Y., Faulkner G.E., O’Brien D.C., Collins S. (2015). Optical receiver front end for optically powered smart dust. Int. J. Circ. Theor. Appl..

[9] Ramaian C.P., Vinayagam N., Ramanathan K.C., Dhanraj J.A., Selvaraju N., Solomon J.M., Anaimuthu S. (2023). A critical evaluation on design and development of smart dust sensor for mechatronics applications. AIP Conf. Proc..

[10] Aasen H., Honkavaara E., Lucieer A., Zarco-Tejada P.J. (2018). Quantitative Remote Sensing at Ultra-High Resolution with UAV Spectroscopy: A Review of Sensor Technology, Measurement Procedures, and Data Correction Workflows. Remote Sens..

[11] Ajith V.S., Jolly K.G. (2024). Hybrid deep learning for object detection in drone imagery: A new metaheuristic based model. Multimed. Tools Appl..

[12] Neumann P.P., Hernandez Bennetts V., Lilienthal A.J., Bartholmai M., Schiller J.H. (2013). Gas source localization with a micro-drone using bio-inspired and particle filter-based algorithms. Adv. Robot..

[13] Kumar S.P., Subeesh A., Jyoti B., Mehta C.R., Pakeerathan K. (2023). Applications of Drones in Smart Agriculture. Smart Agriculture for Developing Nations.

[14] Zhang Z., Zhu L. (2023). A Review on Unmanned Aerial Vehicle Remote Sensing: Platforms, Sensors, Data Processing Methods, and Applications. Drones.

[15] Kucharczyk M., Hugenholtz M. (2021). Remote sensing of natural hazard-related disasters with small drones: Global trends, biases, and research opportunities. Remote Sens. Environ..

[16] Syed Mohd Daud S.M., Mohd Yusof M.Y.P., Heo C.C., Khoo L.S., Chainchel Singh M.K., Mahmood M.S., Nawawi H. (2022). Applications of drone in disaster management: A scoping review. Sci. Justice.

[17] Arroyo P., Gómez-Suárez J., Herrero J.L., Lozano J. (2022). Electrochemical gas sensing module combined with Unmanned Aerial Vehicles for air quality monitoring. Sens. Actuators B Chem..

[18] Bouras A., Gutierrez-Galvez A., Burgués J., Bouzid Y., Pardo A., Guiatni M., Marco S. (2023). Concentration Map Reconstruction for Gas Source Location Using Nano Quadcopters: Metal Oxide Semiconductor Sensor Implementation and Indoor Experiments Validation. Measurement.

[19] Neumann P.P., Kohlhoff H., Hüllmann D., Krentel D., Kluge M., Dzierliński M., Lilienthal A.J., Bartholmai M. (2019). Aerial-based gas tomography—From single beams to complex gas distributions. Eur. J. Remote Sens..

[20] Nery E.W., Kubota L.T. (2013). Sensing approaches on paper-based devices: A review. Anal. Bioanal. Chem..

[21] Martinez A.W., Phillips S.T., Whitesides G.M., Carrilho E. (2010). Diagnostics for the Developing World: Microfluidic Paper-Based Analytical Devices. Anal. Chem..

[22] Singh A.T., Lantigua D., Meka A., Taing S., Pandher M., Camci-Unal G. (2018). Paper-Based Sensors: Emerging Themes and Applications. Sensors.

[23] D’Andrea A., Pomarico G., Nardis S., Paolesse R., Di Natale C., Lvova L. (2019). Chemical Traffic Light: A Self-Calibrating Naked-Eye Sensor for Fluoride. J. Porphyr. Phthalocyanines.

[24] De Meyer T., Hemelsoet K., Van Speybroeck V., De Clerck K. (2014). Substituent effects on absorption spectra of pH indicators: An experimental and computational study of sulfonphthaleine dyes. Dye. Pigment..

[25] Magnaghi L.R., Zanoni C., Alberti G., Biesuz R. (2023). The colorful world of sulfonephthaleins: Current applications in analytical chemistry for “old but gold” molecules. Anal. Chim. Acta.

[26] Hudson-Heck E., Byrne R. (2019). Purification and characterization of thymol blue for spectrophotometric pH measurements in rivers, estuaries, and oceans. Anal. Chim. Acta.

[27] Yimkosol W., Dangkulwanich M. (2021). Finding the pKa Values of a Double-Range Indicator Thymol Blue in a Remote Learning Activity. J. Chem. Educ..

[28] Balderas-Hernández P., Ramírez M.T., Rojas-Hernández A., Gutiérrez A. (1998). Determination of pKa’s for thymol blue in aqueous medium: Evidence of dimer formation. Talanta.

[29] Samayoa-Oviedo H., Mehnert S., Espenship M.F., Weigand M., Laskin J. (2023). Measurement of the Speciation Diagram of Thymol Blue Using Spectrophotometry. J. Chem. Educ..

[30] Basheer M.P., Grattan K.T.V., Sun T., Long A.E., McPolin D., Xie W. (2004). Fiber optic chemical sensor systems for monitoring pH changes in concrete. Advanced Environmental Chemical and Biological Sensing Technologies II.

[31] Buckley R.R., Giorgianni E.J., Luo M.R. (2016). CIELAB for Color Image Encoding (CIELAB, 8-Bit; Domain and Range, Uses). Encyclopedia of Color Science and Technology.

[32] Cebrián P., Pérez-Sienes L., Sanz-Vicente I., López-Molinero Á., de Marcos S., Galbán J. (2022). Solving Color Reproducibility between Digital Devices: A Robust Approach of Smartphones Color Management for Chemical (Bio)Sensors. Biosensors.

[33] Cheng H.D., Jiang X.H., Sun Y., Wang J. (2001). Color Image Segmentation: Advances and Prospects. Pattern Recognit..

[34] Kang H.-C., Han H.-N., Bae H.-C., Kim M.-G., Son J.-Y., Kim Y.-K. (2021). HSV Color-Space-Based Automated Object Localization for Robot Grasping without Prior Knowledge. Appl. Sci..

[35] Moreira G., Magalhães S.A., Pinho T., dos Santos F.N., Cunha M. (2022). Benchmark of Deep Learning and a Proposed HSV Colour Space Models for the Detection and Classification of Greenhouse Tomato. Agronomy.

[36] Bradley D., Roth G. (2007). Adaptive Thresholding using the Integral Image. J. Graph. Tools.

[37] Golilarz N.A., Gao H., Pirasteh S., Yazdi M., Zhou J., Fu Y. (2021). Satellite Multispectral and Hyperspectral Image De-Noising with Enhanced Adaptive Generalized Gaussian Distribution Threshold in the Wavelet Domain. Remote Sens..

[38] Hu Y., Ren J., Yang J., Bai R., Liu J. (2021). Noise reduction by adaptive-SIN filtering for retinal OCT images. Sci. Rep..

[39] Guo H., Yin H., Song S., Zhu X., Ren D. (2024). Application of density clustering with noise combined with particle swarm optimization in UWB indoor positioning. Sci. Rep..

[40] Peng D., Gui Z., Wang D., Ma Y., Huang Z., Zhou Y., Wu H. (2022). Clustering by measuring local direction centrality for data with heterogeneous density and weak connectivity. Nat. Commun..

[41] DJI Zenmuse H20 Series. https://enterprise.dji.com/de/zenmuse-h20-series.

[42] Zhang N., Liu P., Yi Y., Gibril M.E., Wang S., Kong F. (2021). Application of Polyvinyl Acetate/Lignin Copolymer as Bio-Based Coating Material and Its Effects on Paper Properties. Coatings.

[43] Saengdee P., Nuanthong T., Chamnan P., Pattamang P., Thongsook O., Meananeatra R., Ranron N., Pankong K., Uahchinkul W., Jeamsaksiri W. (2022). Development of Starch-Polyvinyl Alcohol Films-based pH indicator for Detection of Penicillin G Residue in Raw Milk. J. Phys. Conf. Ser..

[44] Hou X., Zhao H., Zhang K.-Q., Meng K. (2022). Preparation of Wide-Domain pH Color-Changing Nanocapsules and Application in Hydrogel Fibers. Materials.

[45] Kossyvaki D., Contardi M., Athanassiou A., Fragouli D. (2022). Colorimetric Indicators Based on Anthocyanin Polymer Composites: A Review. Polymers.

[46] Bhowmik N., Barker J.W., Gaus Y.F.A., Breckon T.P. (2022). Lost in Compression: The Impact of Lossy Image Compression on Variable Size Object Detection within Infrared Imagery. arXiv.

[47] Gandor T., Nalepa J. (2022). First Gradually Then Suddenly: Understanding the Impact of Image Compression on Object Detection Using Deep Learning. Sensors.

[48] Sieberth T., Wackrow R., Chandler J.H. (2016). Automatic detection of blurred images in UAV image sets. ISPRS J. Photogramm. Remote Sens..

[49] Kim H., Hyun C.-U., Park H.-D., Cha J. (2023). Image Mapping Accuracy Evaluation Using UAV with Standalone Differential (RTK) and PPP GNSS Positioning Techniques in an Abandoned Mine Site. Sensors.

[50] Canty M.J. (2019). Image Analysis, Classification and Change Detection in Remote Sensing: With Algorithms for Python.

[51] Chityala R., Pudipeddi S. (2020). Image Processing and Acquisition Using Python.

[52] Chakraborty G., Bhattarai A., De R. (2022). Polyelectrolyte–Dye Interactions: An Overview. Polymers.

